# Analysis of characteristic genes and ceRNA regulation mechanism of endometriosis based on full transcriptional sequencing

**DOI:** 10.3389/fgene.2022.902329

**Published:** 2022-07-22

**Authors:** Chengmao Xie, Ziran Yin, Yong Liu

**Affiliations:** Department of Gynecology, Beijing Obstetrics and Gynecology Hospital, Capital Medical University, Beijing Maternal and Child Health Care Hospital, Beijing, China

**Keywords:** endometriosis, whole transcriptome, characteristic genes, immune infiltration, ceRNA

## Abstract

**Background:** Endometriosis is a common gynecological disorder that usually causes infertility, pelvic pain, and ovarian masses. This study aimed to mine the characteristic genes of endometriosis, and explore the regulatory mechanism and potential therapeutic drugs based on whole transcriptome sequencing data and resources from public databases, providing a theoretical basis for the diagnosis and treatment of endometriosis.

**Methods:** The transcriptome data of the five eutopic (EU) and ectopic (EC) endometrium samples were obtained from Beijing Obstetrics and Gynecology Hospital, Beijing, China, and dinified as the own data set. The expression and clinical data of EC and EU samples in GSE25628 and GSE7305 datasets were obtained from the GEO database (https://www.ncbi.nlm.nih.gov/gds). Differential gene expression analysis and weighted gene co-expression network analysis (WGCNA) were used to identify the endometriosis-related differentially expressed genes. Gene Ontology (GO) and Kyoto Encyclopedia of Genes and Genomes (KEGG) pathway enrichment analyses were conducted by the “clusterProfiler” R package. Then, characteristic genes for endometriosis were identified by the least absolute shrinkage and selection operator (LASSO) and support vector machine recursive feature elimination (SVM-RFE) algorithm. The expression of characteristic genes was verified by quantitative reverse transcription polymerase chain reaction (qRT-PCR) and western-blot. The receiver operating characteristic (ROC) curve was used to evaluate the discriminatory ability of characteristic genes. We assessed the abundance of infiltrating immune cells in each sample using MCP-counter and ImmuCellAI algorithms. The competitive endogenous RNA (ceRNA) regulatory network of characteristic genes was created by Cytoscape and potential targeting drugs were obtained in the CTD database.

**Results:** 44 endometriosis-related differentially expressed genes were obtained from GSE25628 and the own dataset. Subsequently, LASSO and SVM-RFE algorithms identified four characteristic genes, namely ACLY, PTGFR, ADH1B, and MYOM1. The results of RT-PCR and western-blot were consistent with those of sequencing. The result of ROC curves indicated that the characteristic genes had powerful abilities in distinguishing EC samples from EU samples. Infiltrating immune cells analysis suggested that there was a certain difference in immune microenvironment between EC and EU samples. The characteristic genes were significantly correlated with specific differential immune cells between EC and EU samples. Then, a ceRNA regulatory network of characteristic genes was constructed and showed a total of 7, 11, 11, and 1 miRNA associated with ACLY, ADH1B, PTGFR, and MYOM1, respectively. Finally, we constructed a gene-compound network and mined 30 drugs targeting ACLY, 33 drugs targeting ADH1B, 13 drugs targeting MYOM1, and 12 drugs targeting PTGFR.

**Conclusion:** Comprehensive bioinformatic analysis was used to identify characteristic genes, and explore ceRNA regulatory network and potential therapeutic agents for endometriosis. Altogether, these findings provide new insights into the diagnosis and treatment of endometriosis.

## Introduction

Endometriosis (EMS) is defined as the presence of ectopic endometrial glands and stroma outside of the uterine cavity and affects 6%–10% of reproductive-aged women. Women with EMS can have symptoms of dyspareunia, dysmenorrhea, irregular uterine bleeding, and chronic pelvic pain (Burney and Giudice, 2012; Johnson et al., 2017; Rogers et al., 2017). Decreased quality of life, increased surgical intervention, and increased use of assisted reproductive technology caused by EMS result in high social costs (Vitagliano et al., 2016). Thus, EMS has become a critical social problem that needs to be addressed. Although medical therapies can relieve symptoms in up to 50%–80% of cases, residual symptoms are still present in at least 20% of patients (Bonocher et al., 2014; Dunselman et al., 2014; Buggio et al., 2017; Parasar et al., 2017). Therefore, exploring new characteristics and potential mechanisms will contribute to the clinical diagnosis of endometriosis and the development of effective treatment methods.

Although there are increasing numbers of studies on EMS immune regulation, its specific mechanism remains unclear. Studies have shown that abnormal immune system function may also be one of the pathogeneses of endometriosis (Seli and Arici, 2003; Symons et al., 2018). The function of almost all types of immune cells in patients with endometriosis is abnormal, that is, the decrease of T cell response activity and NK cytotoxicity, the increase of polyclonal activation and antibody production of B cells, the increase of the number and activation of peritoneal macrophages, the change of inflammatory mediators and so on (Seli and Arici, 2003; Symons et al., 2018). However, so far, there are few studies on the potential genes related to immune cells in endometriosis.

The competitive endogenous RNA (ceRNA) is a novel mode of gene expression regulation, involving lncRNA, microRNA, and mRNA. Through competitive miRNA binding reaction elements (MREs), they form a huge ceRNA regulatory network, enrich the RNA-RNA interaction mechanism and play an important role in the occurrence and development of diseases. LncRNAs are considered to be the main component of ceRNA and regulate the expression level of target genes through competitive binding miRNA. Wang and Chang (2011) reported that lncRNA can regulate gene expression at different levels such as epigenetic, transcriptional, and post transcriptional. Salmena et al. (2011) showed that lncRNAs can act as a natural miRNA “sponge” to competitively bind miRNA through common MREs, inhibit the role of miRNA, and indirectly regulate the expression of target genes. In the ceRNA regulation mode, mRNA and ceRNA have a common MREs binding series, which affects the role of multiple miRNAs, and each ceRNA molecule can be regulated together with multiple different miRNAs to form a huge regulatory network (Chiu et al., 2018). So, exploring the ceRNA regulation mechanism of characteristic genes of endometriosis will provide direction for further study of the molecular mechanism of characteristic genes in the occurrence of endometriosis.

In this study, we screened four characteristic genes of endometriosis (ACLY, PTGFR, ADH1B, and MYOM1) by using the full transcriptome sequencing data of clinical samples and public database resources. Moreover, we further explored the ceRNA regulation mechanism and potential targeted drugs, which provided new insights for exploring the molecular mechanism, clinical diagnosis, and treatment of endometriosis.

## Materials and methods

### Sample collection and processing

Endometriosis or matched control endometrium from the same patients were obtained from Beijing Obstetrics and Gynecology Hospital, Beijing, China. All donors had not taken drugs and hormones before surgery. All tissue samples were taken from tissues discarded during surgery after being approved and informed by the ethics review committee of our hospital.

### Total RNA extraction and whole transcriptome sequencing

In this study, the whole transcriptome data of the five eutopic (EU) and ectopic (EC) endometrium samples were defined as the own data set. RNA was extracted from snap-frozen tissues, which were placed in precooled TRIzol (Thermo Fisher Scientific, Waltham, MA, United States) reagent. Then, RNA was extracted immediately using chloroform extraction and isopropanol precipitation and quantified using a spectrophotometer. A reverse transcription reaction was carried out according to the manufacturer’s instructions in a 20 μl reaction containing 2 μg of total RNA in a gradient cycler (Thermo Fisher Scientific, Waltham, MA, United States). The purity of RNA (OD260/280, OD260/230 ratio) was determined by the NanoDrop ™ One/Onec detection and the RNA integrity (RIN value) was detected by the Agilent 4200 TapeStation system. After the samples passed the test, the library of RNA samples was constructed, and the fragment size of the library was accurately detected by Agilent 4200 tapestation test. After passing the library inspection, different libraries were pooled according to the requirements of effective concentration and target offline data volume, and then Illumina PE150 was sequenced.

### Data source from public database

The GSE25628 dataset containing nine EU and seven EC samples was downloaded from the GEO database (https://www.ncbi.nlm.nih.gov/gds) for WGCNA analysis. The GSE7305 dataset including ten EU and ten EC samples was also mined from the GEO database for gene expression validation.

### Identification of differentially expressed genes

We performed differential expression analysis on five EC and five EU samples from the own dataset by “limma” R package (Ritchie et al., 2015; Phipson et al., 2016). The screening criteria for differentially expressed genes (DEGs) were |log_2_FoldChang (FC)| > 1 and *p*-value < 0.05.

### Weighted gene co-expression network analysis

The expression data of GSE25628 cohort was analyzed using the Weighted gene co-expression network analysis (“WGCNA”) R package to construct the co-expression network which utilized the EC and EU samples as clinical traits (Langfelder and Horvath, 2008). The “goodSamplesGenes” function was used to conduct sample clustering to identify and remove outliers. For making the co-expression network contented the distribution of scale-free network, a soft-thresholding power was computed with the pickSoftThreshold function. The dynamic tree cutting method was used to identify different modules with the minimum number of genes in each module was 100. Subsequently, a merging threshold of 0.2 was set to merge similar modules. The correlation between these modules and clinical traits was further analyzed. Finally, the module with the |correlation coefficient| > 0.5 and *p*-value < 0.05 with was chosen as key modules, in which genes with |MM| > 0.8 and |GS| > 0.6 were identified as key module genes, namely endometriosis-related genes. MM represented the correlation of the gene in the module with the module and GS denoted the correlation of the gene with the trait.

### Functional enrichment analysis

GO and KEGG enrichment analysis was implemented by the “clusterProfiler” R package (Yu et al., 2012). The threshold for significance was *p*-value < 0.05. GO enrichment analysis mainly described the biological processes (BP), cellular components (CC), and molecular functions (MF) correlated with genes. KEGG pathway analysis revealed biological pathways associated with genes.

### Screening for characteristic genes by machine learning

The “glmnet” R package was employed to screen for characteristic genes using the least absolute shrinkage and selection operator (LASSO) regression (Friedman et al., 2010). The area under the receiver operating characteristic (ROC) curve was used to assess the diagnostic sensitivity and specificity of the LASSO model. Support vector machine-recursive feature elimination (SVM-RFE) is a machine learning method in terms of support vector machine algorithms, which can effectively derive a subset of informative genes and make the classification more reliable (Huang et al., 2014). The characteristic genes were filtered out by the “e1071” R package with the ten-fold cross-validation method. Eventually, characteristic genes, namely, potential biomarkers, were determined by overlapping the genes identified by LASSO and SVM-RFE. The classification performance of these potential biomarkers between EC and EU samples was evaluated using the area under the ROC curve, which was drawn by “pROC” R package (Robin et al., 2011).

### Verification of the expression of characteristic genes

EU or EC from the same patients were obtained from Beijing Obstetrics and Gynecology Hospital, Beijing, China. We collected 30 cases were diagnosed as ovarian endometriosis cyst with the inclusion conditions: aged between 20 and 45 years, BMI of 19–25 kg/m^2^, non vegetarian patients, no operative contraindication; The exclusion criteria were diabetes and other endocrine diseases, as well as serious gastrointestinal, cardiopulmonary and liver diseases. All donors had not taken drugs and hormones before surgery, and underwent combined uterine and abdominal surgery because of abnormal uterine bleeding at the same time. All tissue samples were taken from tissues excised during surgery after being approved and informed by patients and the ethics review committee of our hospital. Protein preparation, western blotting, RNA isolation, and qRT-PCR were performed as described previously (Chengmao et al., 2017). The PCR reactions contained 3 mM MgCl_2_, 0.4 μM forward and reverse primers, and 1 μl of LightCycler DNA Master SYBR Green I (10×concentrate, Roche). An initial denaturation step of 95°C for 75 s was performed to activate the FastStart DNA Polymerase and to ensure complete denaturation of the cDNA before amplification. Amplification of ACLY, PTGFR, ADH1B, MYOM1, and GAPDH involved 40 cycles of denaturation at 95°C for 15 s, annealing at 62°C for 10 s, and elongation at 72°C for 25 s. After the last cycle, the amplified products underwent melting curve analysis to check the amplification integrity. Data were analyzed with the Second Derivate Maximum Method using LightCycler Relative Quantification Software. The relative expression level of ACLY, PTGFR, ADH1B, and MYOM1 was normalized to the endogenous control GAPDH. After RT-PCR, we verified the mRNA specificity of ACLY, PTGFR, ADH1B, and MYOM1 with agarose gel electrophoresis. The following primers were used for RT-PCR: ACLY, sense 5′-GAAGGGAGTGAC CATCATCG-3′ and anti-sense 5′-TTA​AAG​CAC​CCA​GGC​TTG​AT-3′; PTGFR, sense 5′-GTT​TTC​CGT​CTG​GCA​GGT​TCT -3′ and anti-sense 5′-AGATGACTTGAGTGGT TGGCTTTT-3′; ADH1B, sense 5′-AGG​GGG​CTG​TTT​ATG​GTG​G-3′ and anti-sense 5′-GGT​ACG​GAT​ACT​TTT​CCC​AGA​GT-3′; MYOM1, sense 5′-CCGAGACATATCA TGCCAAGC-3′ and anti-sense 5′-CGT​GTG​GGA​GCG​AGG​TTT​AAT-3′. Proteins were extracted using the M-PEK kit, following the manufacturer’s instructions. The protein concentration in the extracts was determined using the Quick Start Bradford protein assay. SDS-PAGE was conducted with protein samples of approximately 20 μg loaded onto a 7% Tris-acetate gel, run at 120 V for 2 h. To maintain the integrity of the target proteins, samples were not heated prior to electrophoresis. Proteins in the gel were transferred onto an Immobilon-NC transfer membrane at 300 mA for 90 min. The membrane was blocked in 5% nonfat milk powder in Tris-buffered saline with 0.1% Tween 20 (TBST) for 2 h, incubated overnight at 4°C with rabbit monoclonal anti-ATP citrate lyase (*ACLY*) (ab40793; Abcam), mouse monoclonal anti-alcohol dehydrogenase1B (*ADH1B*) (Cat No. 66939-1-Ig; Proteintech), rabbit monoclonal anti-Myomesin1 (*MYOM1*) (ab201228; Abcam) and rabbit monoclonal anti- Prostaglandin F2 alpha Receptor/PTGFR (*PTGFR*) (ab126709; Abcam) antibodies (1:1000 in TBST), and washed with TBST three times for 10 min each. It was then incubated for 45 min at room temperature (RT) with a goat anti-rabbit IgG and a mouse anti-rabbit IgG-horseradish peroxidase (HRP)-conjugated secondary antibody (1:5000 in TBST). After three 10-min washes of the membrane in TBST, the signal was recorded by digital imaging using ChemiDocTM XRS+ with Image Lab™ Software (BIO-RAD, Hercules, California, United States). β-actin served as an internal control.

### Immune infiltration analysis

Infiltrating immune cells in the endometrium samples were evaluated by the MCP-counter and ImmuCellAI algorithms. MCP-counter was implemented with the “MCP-counter” R package to compute the content of eight types of immune cells, fibroblasts, and endothelial cells (Becht et al., 2016). ImmuCellAI online website (http://bioinfo.life. hust edu.cn/web/ImmuCellAI) was applied to estimate the proportions of 18 T cells and six other types of immune cells (B cells, NK cells, Monocyte cells, Macrophage cells, Neutrophil cells, and DC cells) (Miao et al., 2020).

### Construction of ceRNA network

Aiming to construct the ceRNA regulatory network for the characteristic genes, we first screened the differentially expressed miRNAs (DE-miRNAs) and lncRNAs (DE-lncRNAs) between EU and EC samples using whole transcriptome data from the own dataset, with |log_2_FoldChange (FC)| > 1 and *p*-value < 0.05 as screening criteria. Then, we predicted the miRNAs targeting the characteristic genes using the miRWalk database (http://mirwalk.umm.uni-heidelberg.de/). The starbase database (http://starbase.sysu.edu.cn/) was exploited to predict the lncRNAs targeting the corresponding miRNAs. Finally, we selected the lncRNA-mRNA pairs with opposite expression trends and miRNA-mRNA pairs with opposite expression trends to establish the ceRNA regulatory network of the characteristic genes. The network was visualized using Cytoscape software (Shannon et al., 2003).

### Prediction of potential drugs targeting the characteristic genes

The CTD database (http://ctdbase.org/) was utilized to predict the potential drugs targeting characteristic genes. The network of characteristic gene-compound was visualized using Cytoscape software (Shannon et al., 2003).

### Statistical analysis

All bioinformatics analyses were conducted using the R programming language, and the data from different groups were compared by the Wilcoxon test. If not specified above, a *p*-value less than 0.05 was considered statistically significant.

## Results

### Endometriosis-related differentially expressed genes

**FIGURE 1:**
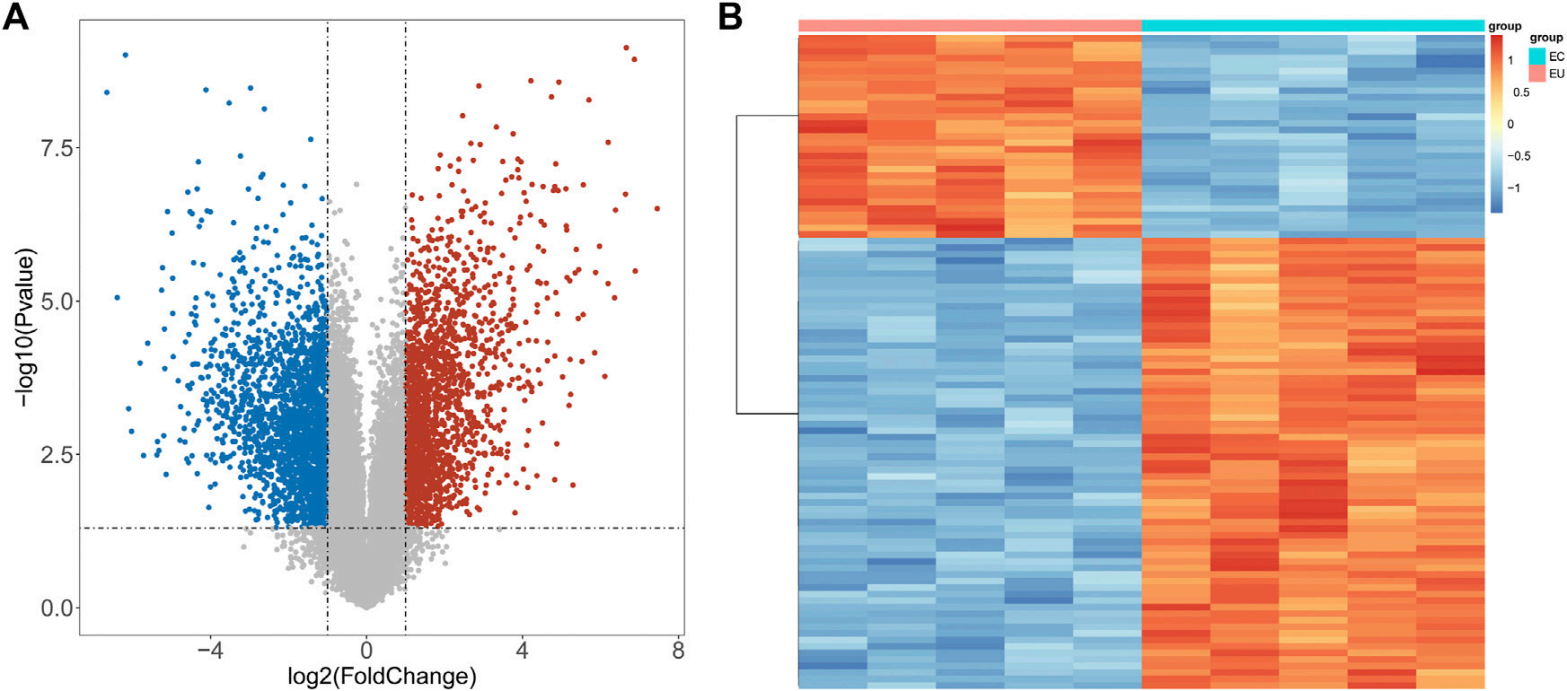
DEGs between EU and EC samples. **(A)** Volcano map of DEGs: The red dot indicates that the gene expression is upregulated, the blue dot indicates that the gene expression is down regulated, and the gray dot indicates that there is no significant difference between these genes. **(B)** Heat map of top100 DEGs: Each small square indicates each gene, and its color indicates the expression amount of the gene. The greater the expression amount, the darker the color (red indicates high expression and blue indicates low expression).

**FIGURE 2:**
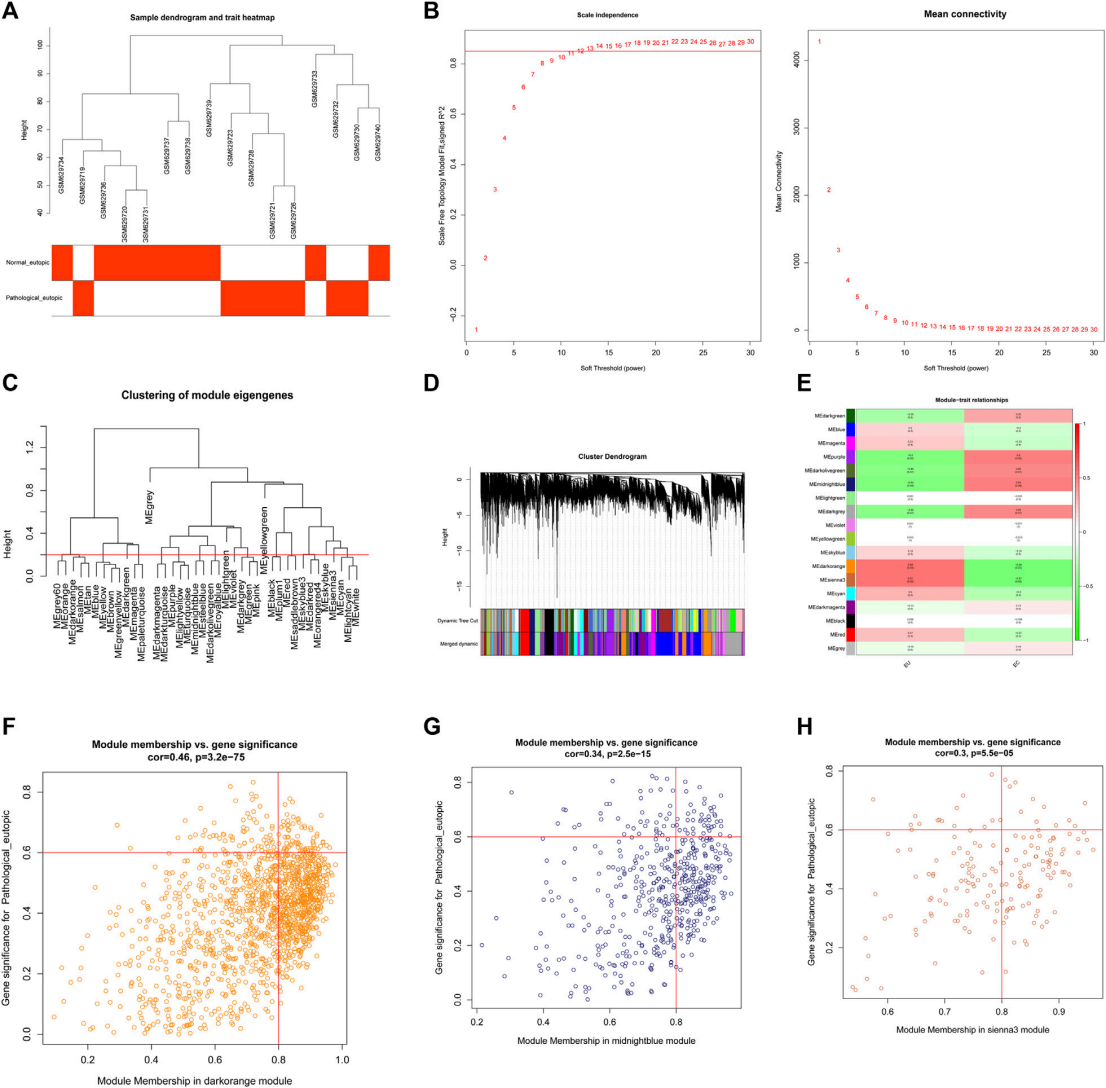
Construction and module analysis of weighted gene co-expression network analysis (WGCNA). **(A)** Clustering and phenotypic information of combined data samples, the upper part of the figure is clustering, and the lower part is phenotype. The color represents disease. **(B)** Determination of the soft threshold. **(C)** Merging of the similar modules analyzed by the dynamic cutting tree algorithm. **(D)** Clustering dendrogram of genes with different similarities based on topological overlap and the assigned module color. **(E)** The heat map of correlation between different modules and clinical characters. The ordinate represents different modules and the abscissa represents different traits. Each block represents the correlation coefficient and significance *p* value of a module and a trait. **(F)** Module membership in sienna3 module, the vertical line is |MM| = 0.6 and the horizontal line is |GS| = 0.8. The key gene of the module we selected is in the box of the upper right corner of the figure. **(G)** Module membership in darkorange module, the vertical line is |MM| = 0.6 and the horizontal line is |GS| = 0.8. The key gene of the module we selected is in the box of the upper right corner of the figure. **(H)** Module membership in midnightblue module, the vertical line is |MM| = 0.6 and the horizontal line is |GS| = 0.8. The key gene of the module we selected is in the box of the upper right corner of the figure.

**FIGURE 3:**
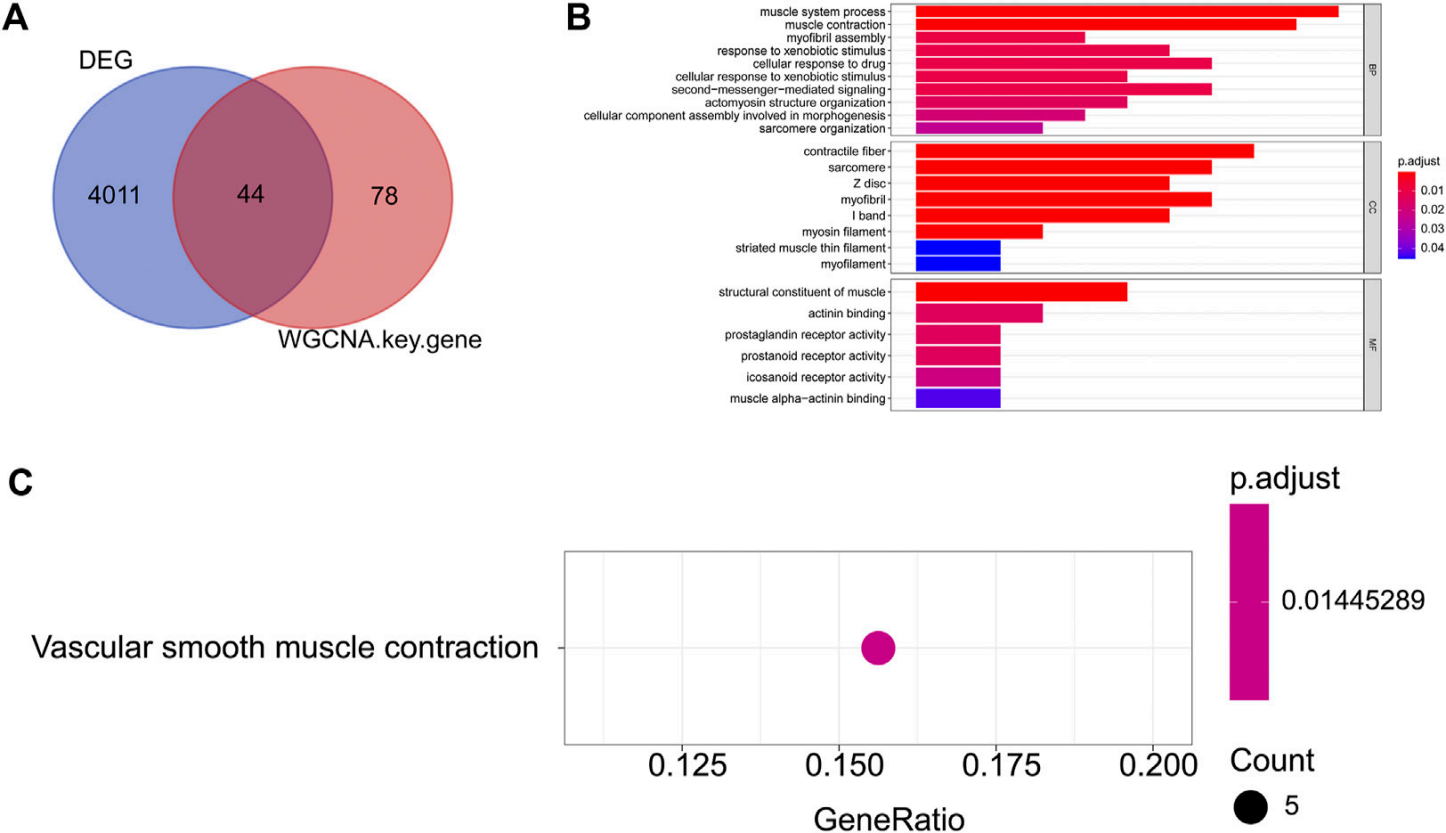
GO and KEGG enrichment of endometriosis-related DEGs **(A)** The Venn diagram of the DEGs and key module genes were identified by WGCNA; **(B)** TOP10 GO (BP), GO (CC), and GO (MF) entries. The ordinate represents each GO term, which is sorted according to the *p*-value. The abscissa represents the gene proportion, the color of the column represents the *p*-value, the more reder represents the higher reliability, the length of the column represents the number of genes involved, and the longer the column length represents the greater number of genes involved; **(C)** KEGG enrichment result. The ordinate represents each KEGG term. The abscissa represents the gene proportion.

To explore the regulatory mechanisms of endometriosis, we conducted whole transcriptome sequencing on five pairs of collected EC and EU samples using high-throughput sequencing technology. Based on the sequencing data, we detected 4057 DEGs in EC samples compared to EU samples using the “limma” package, including 1842 upregulated genes and 2215 downregulated genes (Figure 1A, Supplementary Table S1). Top100 DEGs were displayed in a heatmap (Figure 1B). To further mine the genes associated with endometriosis, we downloaded the data of GSE25628 dataset from the GEO database and performed WGCNA. No obvious outliers were excluded by cluster analysis (Figure 2A). 13 was selected as the optimal soft threshold with an *R* ^2^ = 0.85 (Figure 2B). Based on the optimal soft threshold, we divided the genes into different modules according to dynamic tree cutting algorithm. After merging, a total of 18 modules were generated (Figures 2C,D). Correlations between modules and clinical traits (EU samples and EC samples) were calculated (Figure 2E). Three modules (sienna3, darkorange, and midnightblue) was considered as the key modules as the |correlation coefficient| > 0.5 and *p*-value < 0.05 (Figure 2E). According to the criterion of |MM| > 0.8 and |GS| > 0.6, 11 genes in sienna3 module, 69 genes in darkorange module, and 41 genes in midnightblue module were authenticated as key module genes, namely endometriosis-related genes (Figures 2F–H). Hence, 44 endometriosis-related DEGs were obtained by taking the intersection of DEGs and key module genes (Figure 3A, Supplementary Table S2).

### Function of endometriosis-related differentially expressed genes

To further understand the biological functions and the pathways involved in the endometriosis-related DEGs, we conducted GO and KEGG functional enrichment analysis. A total of 49 GO entries (including 35 BP entries, 8 CC entries, and 6 MF entries) and one KEGG pathways were enriched (Supplementary Table S3). Top10 GO entries under each category were listed in Figure 3B. We found that these genes were associated with “muscle system process,” “muscle contraction,” “myofibril assembly,” “response to xenobiotic stimulus,” “cellular response to drug,” “second-messenger- mediated signaling,” “positive regulation of inflammatory response,” “cyclic-nucleotide-mediated signaling” and so on. In addition, for KEGG pathways, these 55 genes were enriched for “Vascular smooth muscle contraction” (Figure 3C).

### Characteristic genes for endometriosis

**FIGURE 4:**
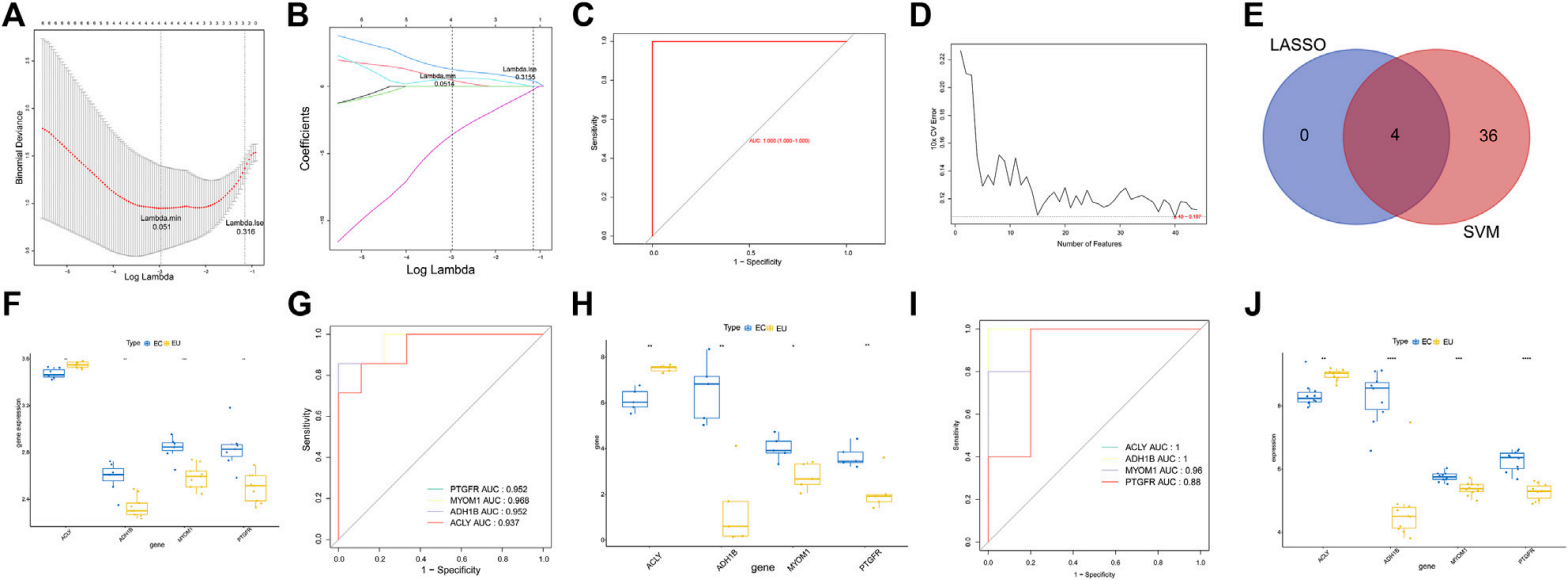
The characteristic genes for endometriosis. **(A)** The penalty diagram of lasso logistic regression coefficient. The abscissa deviance represents the proportion of the residual explained by the model, showing the variation relationship between the number of characteristic genes and the proportion of the explained residual (DEV), and the ordinate is the coefficient of genes. The dotted line position on the left is the position where the cross-validation error is the smallest. Determine the corresponding abscissa log (lambda) according to this position (lambda. Min). The number of characteristic genes is displayed on the top. **(B)** The penalty diagram of lasso logic coefficient. The abscissa is log (lambda), and the ordinate represents the error of cross validation. With the change of the penalty coefficient lambda, the coefficients of most variables are compressed to 0. When the 10-fold cross validation error is the smallest, the best lambda value is selected, and the best lambda is obtained in lambda When min = 0.051, select four non-zero coefficient variables. **(C)** The ROC curve of lasso model. **(D)** The relationship between generalization error and characteristic number. The abscissa represents the number of characteristic genes, and the ordinate represents the generalization error under 10-fold cross validation. The trend of broken line graph represents the relationship between the number of characteristic genes and generalization error. **(E)** The Venn diagram of characteristic genes identified by LASSO and SVM-RFP. **(F)** The box diagram of the expression of characteristic genes in the GSE25628 dataset. **(G)** The ROC curve of the characteristic genes in the GSE25628 dataset. **(H)** The box diagram of the expression of characteristic genes in the own dataset gene. **(I)** The ROC curve of characteristic genes in the own dataset. **(J)** The box plot of the expression of characteristic genes in the GEO7305 dataset. *p < 0.05, **p < 0.01, ***p < 0.001.

To dig the characteristic genes from the 44 endometriosis-related DEGs, we adopted LASSO and SVM-RFE algorithms in the GSE25628 dataset. Four characteristic genes (ACLY, PTGFR, ADH1B, and MYOM1) were discerned using the LASSO algorithm (Figures 4A,B). ROC curve with AUC value was one indicating the LASSO model performed well (Figure 4C). Meanwhile, 40 characteristic genes were selected with the SVM-RFE algorithm, as shown in Figure 4D and Supplementary Table S4. Hence, four genes were defined as characteristic genes for endometriosis by overlapping the genes derived from these two algorithms, including ACLY, PTGFR, ADH1B, and MYOM1 (Figure 4E). In order to investigate the diagnostic ability of each characteristic gene, we plotted the ROC curves for each gene. As shown in Figure 4F, ACLY was down-regulated in EC samples, and ADH1B, MYOM1, and PTGFR were up-regulated in EC samples. The AUC values of these genes were all greater than 0.8, indicating that the characteristic genes could distinguish ECl and EU samples powerfully (Figure 4G). The results were also validated in the own dataset (Figures 4H,I). In addition, we further validated the expression of characteristic genes by external dataset GSE7305. Consistent with the results of GSE25628 dataset and the own dataset, ACLY was down-regulated in EC samples, and ADH1B, MYOM1, and PTGFR were up-regulated in EC samples (Figure 4J). These results indicated that the four characteristic genes have potential diagnostic value in clinical practice.

### Verification of characteristic genes expression

**FIGURE 5:**
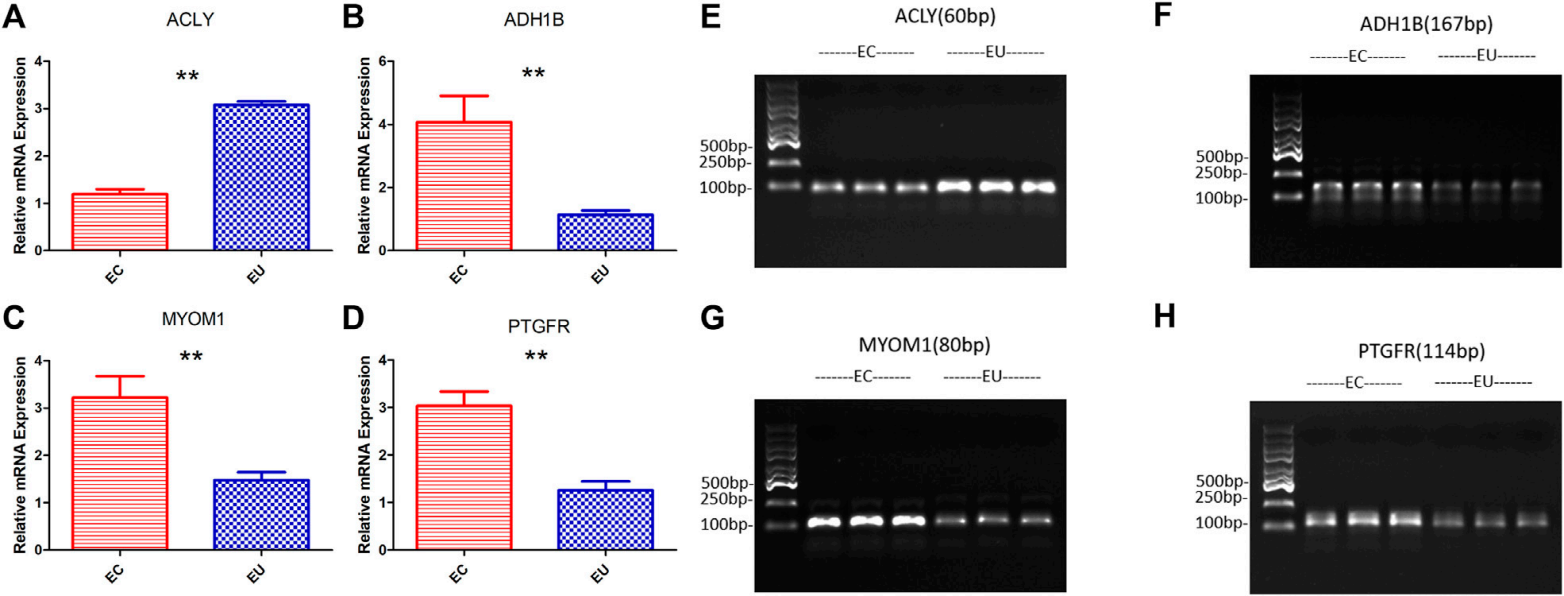
The results of RT-PCR and the graphs (CT curves) to verify the expression of characteristic genes at the mRNA level; **(A)**, **(E)** ACLY; **(B)**, **(F)** ADH1B; **(C)**, **(G)** MYOM1; **(D)**, **(H)** PTGFR; **p < 0.01.

**FIGURE 6:**
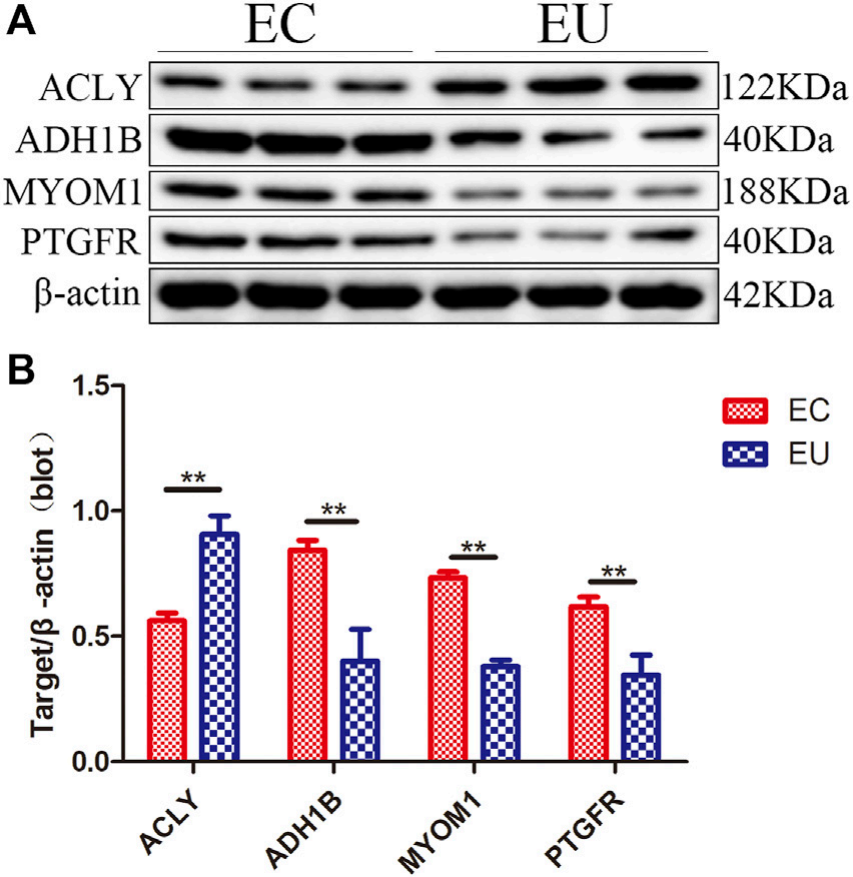
The results of western-blot to verify the expression of characteristic genes at the protein level; **(A)** Western blotting results; **(B)** Densitometry of the western blot; **p < 0.01.

We verified the results of the screened characteristic genes at the mRNA and protein level. The verification results revealed that the mRNA levels of ADH1B (Figure 5B), MYOM1 (Figure 5C), and PTGFR (Figure 5D) in EU were higher than those in the control group, while the results of ACLY (Figure 5A) were the opposite, which consistent with the sequencing data. The qRT-PCR graphs (CT curves) of ACLY, ADH1B, MYOM1, and PTGFR were showed in (Figures 5E–H). The verification results revealed that the protein (Figures 6A,B) levels of ADH1B, MYOM1, and PTGFR in EU were higher than those in the control group, while the results of ACLY were the opposite, consistent with the RT-PCR. The samples derive from the same experiment and those gels/blots were processed in parallel.

### Characteristic genes and infiltrating immune cells

**FIGURE 7:**
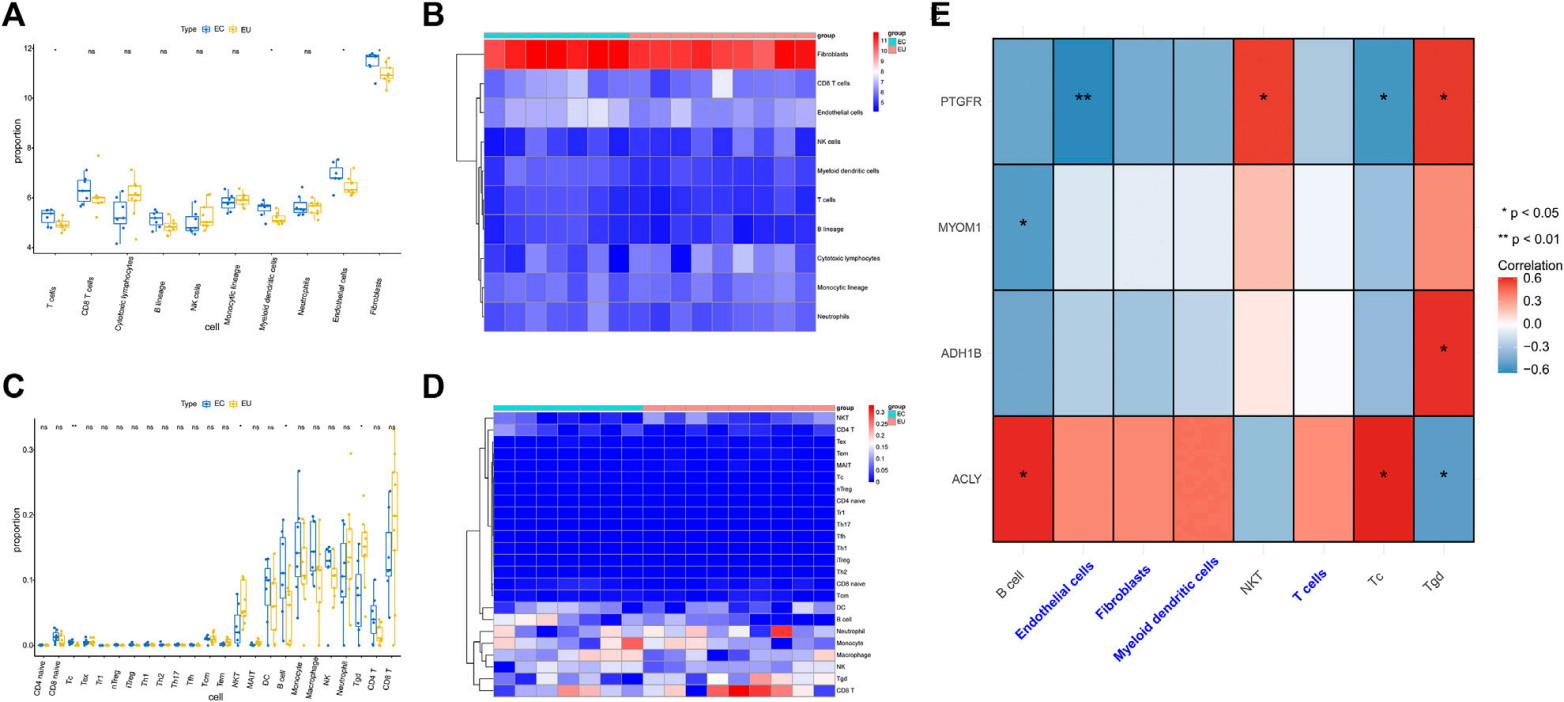
The characteristic genes and infiltrating immune cells. **(A)** The box plot of cell content was calculated by MCP-counter between different EU and EC groups. **(B)** The heat map of different cell contents was calculated by MCP-counter. **(C)** The box diagram of immune cell difference between EU group and EC group was calculated by ImmuneCellAI. **(D)** The heat map of different cell contents was calculated by ImmuneCellAI. **(E)** The heat map showed the correlations between immune cells and the characteristic gene. ceRNA regulatory network for characteristic genes.

In order to evaluate the discrepancy of immune microenvironment between the EC and EU samples, we estimated the enrichment of different immune cells using the MCP-counter and ImmuneCellAI algorithm. The results from MCP-counter showed that the EU samples had significantly higher proportions of T cells, Myeloid dendritic cells, endothelial cells, and fibroblasts (Figures 7A,B). The results from ImmuneCellAI algorithm indicated that the EC samples had significantly lower proportions of NKT cells and Tgd cells, and significantly higher proportions of Tc cells and B cells (Figures 7C,D). These findings suggested that there was a certain difference in immune microenvironment between EU and EC samples.

We further analyzed the spearman correlation between the four characteristic genes and differential immune cells. As shown in Figure 7E and Supplementary Table S5, PTGFR was positively correlated with NKT cells and Tgd cells. PTGFR was negatively correlated with endothelial cells and Tc cells. MYOM was negatively correlated with B cells. ADH1B was positively correlated with Tgd cells. ACLY was positively correlated with B cells and Tc cells. ACLY was negatively correlated with Tgd cells.

**FIGURE 8:**
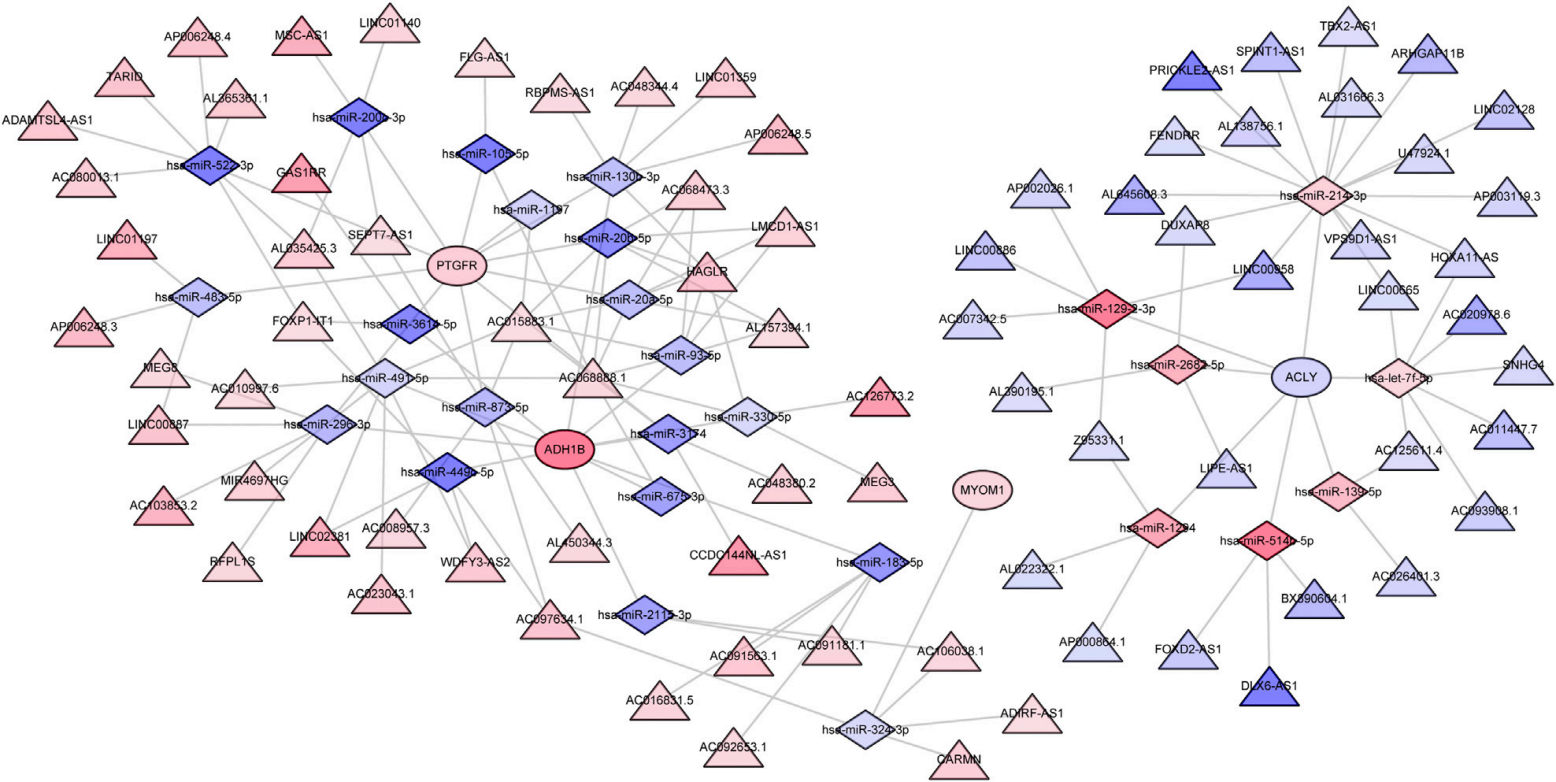
The ceRNA regulatory network for characteristic genes. The circle indicates mRNA; the triangle represents lncRNA and the diamond represents miRNA. The color indicates the expression trend, red indicates upregulation, blue indicates downregulation, and the darker the color, the greater the value of |logFC|. Potential drugs targeting the characteristic genes.

To further explore the regulatory mechanism of characteristic genes in endometriosis, we proceeded with lncRNA-miRNA-mRNA network construction by utilizing whole transcriptome data from the own dataset. Firstly, we identified 295 differentially expressed miRNAs (DE-miRNAs) between EC and EU samples, including 147 upregulated miRNAs and 148 downregulated miRNAs (Supplementary Figure S1A; Supplementary Table S6). Top100 DE-miRNAs were displayed in the heatmap (Supplementary Figure S1B). Meanwhile, 476 differentially expressed lncRNAs (DE-lncRNAs) were extracted from DEGs between EC and EU samples, including 217 upregulated lncRNAs and 259 downregulated lncRNAs. We took the intersection of DE-miRNAs with miRNAs targeting four characteristic genes predicted at the miRWalk database, and retained miRNA-mRNA relationship pairs with opposite expression trends. Next, we intersected the DE-lncRNAs with the lncRNAs targeting the corresponding miRNAs predicted in the starbase database, and retained the lncRNA-miRNA relationship pairs with opposite expression trends. Ultimately, a lncRNA-miRNA-mRNA regulatory network containing 111 nodes (4 mRNAs, 27 miRNAs, and 80 lncRNAs) and 150 edges was generated (Figure 8, Supplementary Table S7).

**FIGURE 9:**
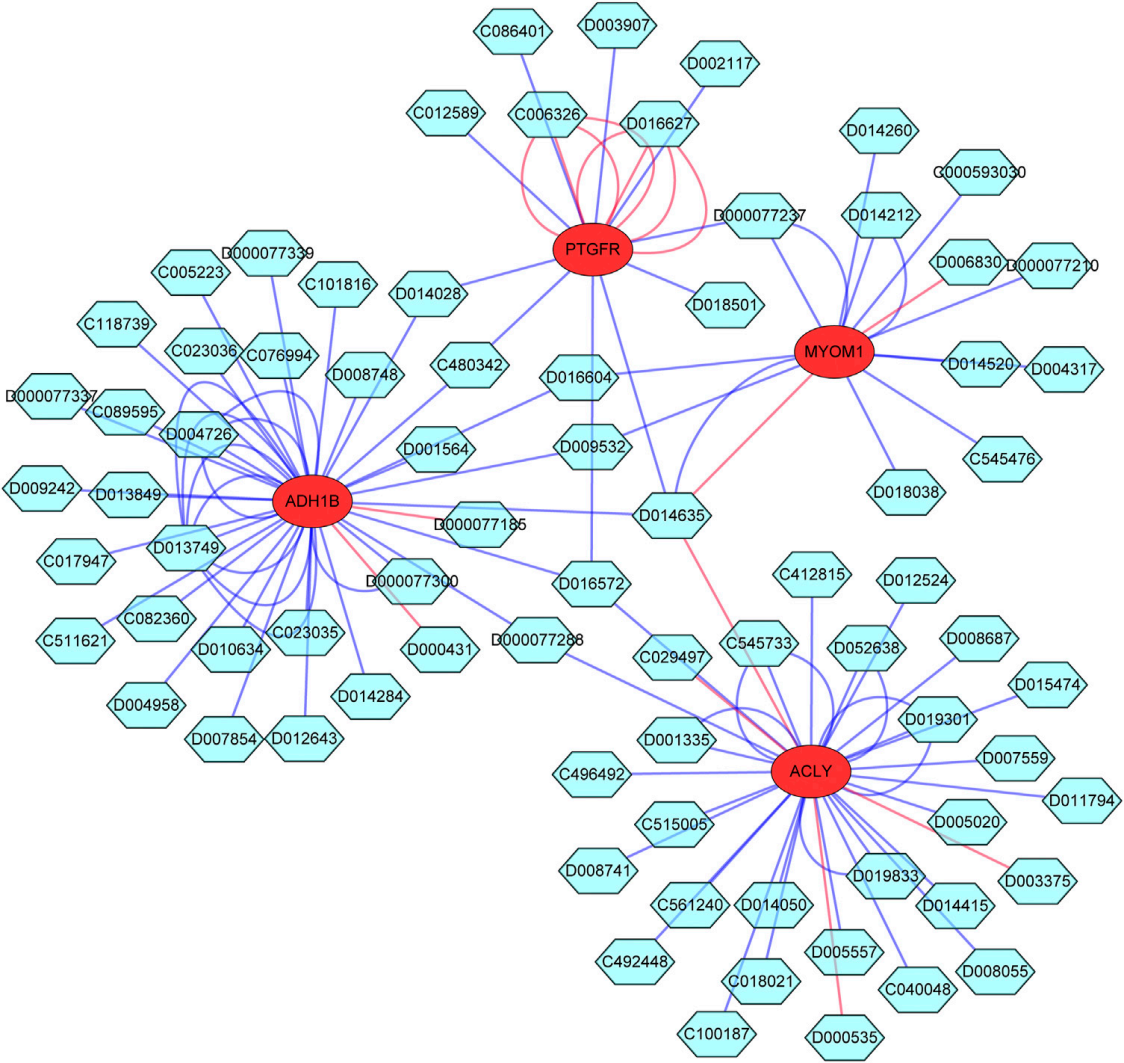
The Potential drugs targeting the characteristic genes. The red circle indicates mRNA; The blue green hexagon indicates drugs; The line color indicates different influence effects, red indicates increased expression, and blue indicates decreased expression.

To explore the potential drugs for endometriosis therapy, we searched the potential drugs targeting the characteristic genes in the CTD database. As shown in Figure 9 and Supplementary Table S8, 30 drugs targeting ACLY, 33 drugs targeting ADH1B, 13 drugs targeting MYOM1, and 12 drugs targeting PTGFR were mined. Finally, a gene-compound network containing 81 nodes (4 mRNAs and 77 compounds) and 115 edges was generated and exhibited in Figure 9. We noted that fatostatin, abrine, cyclosporine, ganoderic acid A, ivermectin, quercetin, troglitazone, and tunicamycin targeted ACLY. Bosentan, cyclosporine, entinostat, estradiol, fipronil, leflunomide, troglitazone, and resveratrol targeted ADH1B. Doxorubicin, GSK-J4, and incobotulinumtoxin A targeted MYOM1. Fluprostenol, oxidopamine, antirheumatic agents, calcitriol, clothianidin, cyclosporine, dexamethasone, and trichostatin A targeted PTGFR.

## Discussion

EMS is a common inflammatory disease, which is caused by the spread or growth of endometrioid tissue in abnormal parts, or by the metaplasia of endometrial tissue outside normal parts, which is characterized by the functional response of glands and stroma to local, endogenous and exogenous hormone stimulation, and its main clinical symptoms are pain, mainly including pelvic pain, infertility Dysmenorrhea and difficult sexual intercourse (Shannon et al., 2003; Acién and Velasco, 2013; Practice Committee of the American Society for Reproductive Medicine, 2014; Peiris et al., 2018; Zondervan et al., 2020). Ectopic endometrium is usually found in pelvic peritoneum and pelvic organs (ovary, fallopian tube, intestine, sigmoid colon and rectum, uterine ligament, rectovaginal septum, and bladder), and can also be planted in tissues and organs outside the pelvic cavity (such as navel, vulva, laparotomy scar, appendix, and lung) (Subramanian and Agarwal, 2010; Rolla, 2019). At present, there are many theories about the pathogenesis of EMS, among which the most recognized is the menstrual blood retrograde theory proposed by Sampson (1928) in the early 20th century, that is, the disease originates from the retrograde menstruation of endometrial tissue, and the mechanical transfer of endometrial tissue during menstruation progresses to the pelvis, resulting in invasive implantation and ectopic growth of endometrial tissue, which develops into endometriosis (Kobayashi et al., 2014). However, this theory cannot fully explain the origin and other aspects of the disease. Therefore, follow-up studies have put forward a variety of hypotheses for the pathophysiology of EMS, such as coelomic metaplasia, cellular immune changes, metastasis, genetic basis, environmental basis, and multifactor genetic model of the interaction between specific genes and the environment, but the exact pathogenesis of EMS is still unclear (Giudice and Kao, 2004).

As far as we know, our study is the first time to screen the characteristic gene of EMS and explore the ceRNA regulatory mechanism by using our own full transcriptome sequencing data of clinical samples and public database resources. 44 endometriosis-related DEGs were authenticated by differential analysis and WGCNA. Then we analyzed the functional enrichment of these genes, which showed that these genes were mainly involved in biological processes such as muscle contraction, muscle fiber assembly, second messenger mediated signal, positive regulation of inflammatory response, and vascular smooth muscle contraction pathway. Inflammatory response is a defensive response caused by injury and dysfunction caused by external stimulation. After the inflammatory response is activated, it stimulates tissue nerve endings by releasing inflammatory factors, so as to participate in the formation of pain (McKinnon et al., 2015). In recent years, a large number of studies have shown that inflammatory response and emergency response participate in the pathological process of EMS (Samimi et al., 2019; Asally et al., 2020; Wei et al., 2020). A variety of inflammatory factors are differentially expressed in EMS patients, which can promote the occurrence of EMS, and also play an important role in the generation of pelvic pain in EMS patients (Ayubi and Safiri, 2017). Then we obtained four characteristic genes (ACLY, PTGFR, ADH1B, and MYOM1) of EMS through machine learning, and verified its effective diagnostic ability through ROC curve analysis. Studies have reported that prostaglandin F2a (PTGFR) is a pain causing substance in the body, which is positively correlated with the degree of dysmenorrhea (McAllister et al., 2016). Lysosome’s rupture and bleed during menstruation, and a large amount of PTGFR release leads to a significant aggravation of local pain (Yoshino et al., 2018). PTGFR also can significantly improve the activity of aromatase P450, which is a key enzyme in the process of estrogen biosynthesis and can stimulate EMS lesions to increase the synthesized estrogen, and then promote the combination of estrogen and its receptor to accelerate the growth of EMS lesions (Kitawaki et al., 1999). In addition, PTGFR can also reduce the expression of MMPs, scavenger receptor and CD36 in peritoneal macrophages, resulting in the backflow endometrial tissue escaping the clearance of the body, and then the three processes of adhesion, invasion and angiogenesis. PTGFR also stimulates the expression of VEGF, induces the proliferation of epithelial cells, and further promotes the occurrence and development of EMS (Chuang et al., 2009). As far as we know, the relationship between ACLY, ADH1B, MYOM1 and EMS has not been reported. This is the first time to show that those genes are the characteristic gene of EMS, which may play an important role in the occurrence of EMS. However, their role and mechanism in the occurrence and development of EMS are not clear, and further research is needed.

By comparing the infiltrating immune cells of eutopic endometrium samples and ectopic endometrium samples, we found that there were some differences between them, and found that the characteristic gene was related to some immune cells. In fact, the various immune cells in the abdominal cavity environment improve the invasive and adhesive abilities of endometrial cells, including dendritic cells, macrophages, mast cells, NK cells, and T cells, which can lead to ectopic endometrium flowing back into the pelvic and abdominal cavities with menstrual blood (Symons et al., 2018). During the menstrual cycle, endometrial-like tissue can spread outside its endometrial location, and these lesions can attract cytotoxic T cells, macrophages, and NK cells (Sampson, 1927; Dmowski et al., 2001; Christodoulakos et al., 2007; Hansen et al., 2010). Subsequently, the activation of the inflammatory response promotes the secretion of cytokines and chemokines in the abdominal cavity to create a microenvironment and induce the development of ectopic endometrial tissue by promoting local angiogenesis and destroying the process of endometrial apoptosis (Mantovani et al., 2008). The large amount of evidence mentioned above as well as our current results show that several types of invasive immune cells have a crucial role in EMS and should be investigated further in future studies.

Noncoding RNA (ncRNA) transcribed from DNA genome but unable to encode protein plays a role as a general regulator in cell process. Generally, ncRNA can be divided into two categories according to its size: small long-chain noncoding RNA (<200 nucleotides in length) and long-chain noncoding RNA (length ≥200 nucleotides). MiRNA is one of the most concerned small ncRNAs, which has been proved to be abnormally expressed in EMS, but the specific mechanism remains to be clarified (Beermann et al., 2016; Saare et al., 2017). As a new star in the progress of RNA sequencing technology, lncRNA has set off a research upsurge in recent years, and EMS is no exception. It is worth noting that the emerging competitive endogenous RNA (ceRNA) hypothesis shows that lncRNA can be used as a miRNA “sponge” to regulate the target mRNA (Panir et al., 2018). This hypothesis has been confirmed in EMS: lncRNAH19, the first reported in EMS, absorbs mirnalet-7 to regulate its downstream gene IGF1R to affect the proliferation of endometrial stromal cells (Salmena et al., 2011). Exosomes secreted by endometrial stromal cells can transmit information to other cells and promote the occurrence and development of EMS (Bonafede et al., 2016; Sun et al., 2018). However, there are few studies on the comprehensive analysis of EMS related miRNA and lncRNA in ceRNA network environment (Ghazal et al., 2015). In the early stage, after obtaining the expression trend of mRNA, miRNA and lncRNA, we constructed the mRNA-miRNA-lncRNA network of characteristic genes based on the sequencing data of EMS, and finally obtained lots kinds of gene signal axes about EMS, which could provide new ideas and potential targets for us to study the mechanism of EMS in the future.

At present, surgery is the main treatment of endometriosis in clinic, but conservative surgery alone is usually difficult to cure, and drug treatment is an important adjuvant treatment, so EMS patients often need long-term drug management after operation. To explore the potential drugs for endometriosis therapy, we searched the potential drugs targeting the characteristic genes in the CTD database. We noted that fatostatin, abrine, cyclosporine, ganoderic acid A, ivermectin, quercetin, troglitazone, and tunicamycin targeted ACLY. Bosentan, cyclosporine, entinostat, estradiol, fipronil, leflunomide, troglitazone, and resveratrol targeted ADH1B. Doxorubicin, GSK-J4, and incobotulinumtoxin A targeted MYOM1. Fluprostenol, oxidopamine, antirheumatic agents, calcitriol, clothianidin, cyclosporine, dexamethasone, and trichostatin A targeted PTGFR. All the above research results can provide theoretical guidance and potential therapeutic targets for the clinical drug treatment of EMS in the future.

In summary, this study digs the characteristic genes of EMS, which provides a basis for the study of the molecular mechanism, clinical diagnosis, and treatment of EMS. However, our research has not further verified the relevant molecular mechanism, which needs to be further improved in the follow-up research.

## Data Availability

The datasets presented in this study can be found in online repositories. The name of the repository and accession number can be found below: NCBI; PRJNA819530.
