# Supplementary material for: Analysis of characteristic genes and ceRNA regulation mechanism of endometriosis based on full transcriptional sequencing
Source: Front Genet. 2022 Jul 22;13:902329. doi: 10.3389/fgene.2022.902329 (PMC9353714; doi:10.3389/fgene.2022.902329)
Supplement: Supplementary file 1 [file DataSheet1.ZIP › Supplementary material/Figure S1.docx]

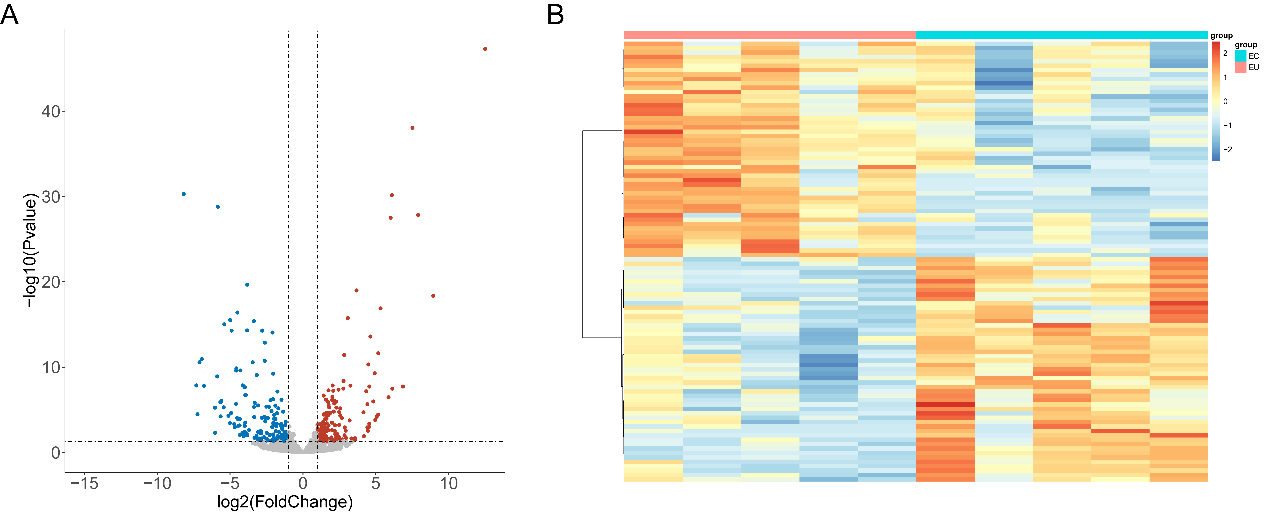


Figure.S1：DE-miRNAs between EU and EC samples in the own dataset. (A) Volcano map of DE-miRNAs: Each dot represents a miRNA, and the blue and red dots represent significantly differentially expressed miRNA. The red dot indicates that the miRNA expression is up-regulated, the blue dot indicates that the miRNA expression is down regulated, and the gray dot indicates that there is no significant difference between these miRNAs. (B) Heat map of TOP100 DE-miRNAs: Each small square indicates each miRNA, and its color indicates the expression amount of the miRNA. The greater the expression amount, the darker the color (red indicates high expression and blue indicates low expression).
