# Supplementary figures and images for: Analysis of characteristic genes and ceRNA regulation mechanism of endometriosis based on full transcriptional sequencing
Source: Front Genet. 2022 Jul 22;13:902329. doi: 10.3389/fgene.2022.902329 (PMC9353714; doi:10.3389/fgene.2022.902329)

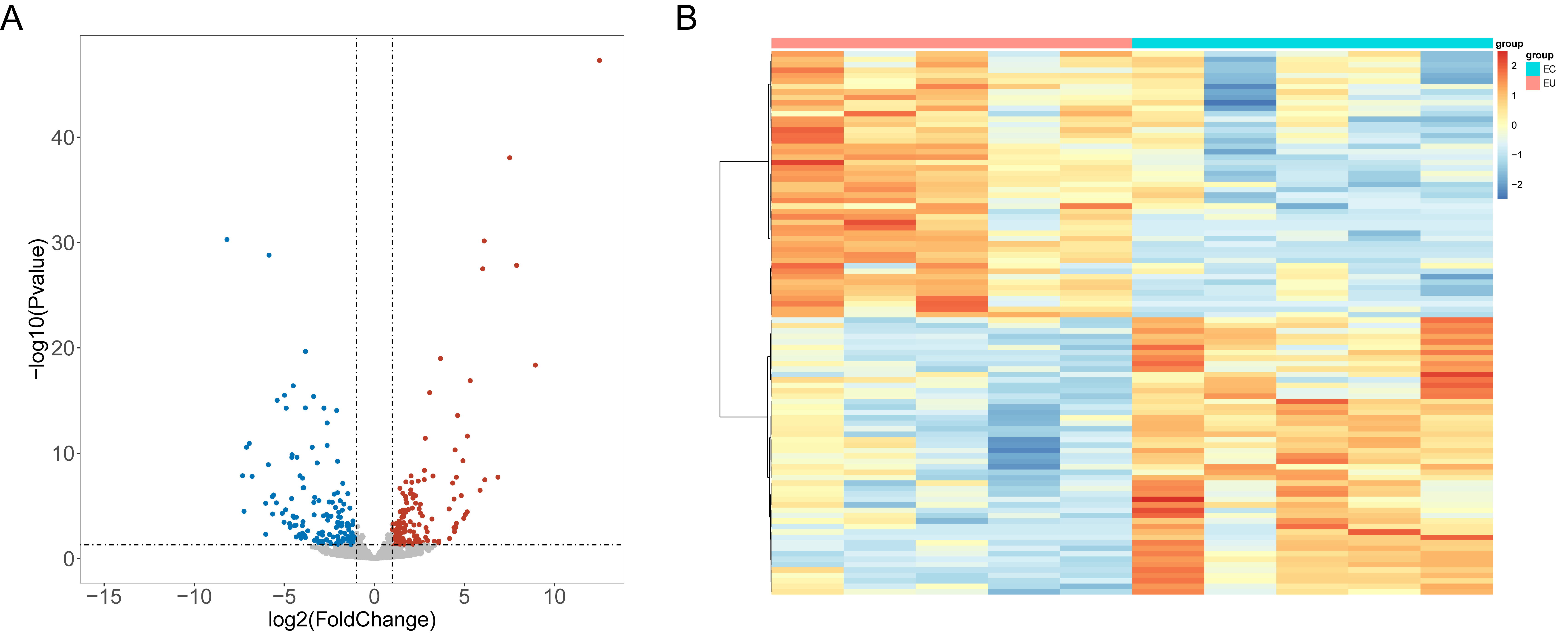

Supplement: Supplementary file 1 [file DataSheet1.ZIP › Supplementary material/Figure S1.tif]
